# Systematic diet composition swap in a mouse genome-scale metabolic model reveals determinants of obesogenic diet metabolism in liver cancer

**DOI:** 10.1016/j.isci.2023.106040

**Published:** 2023-01-24

**Authors:** Frederick Clasen, Patrícia M. Nunes, Gholamreza Bidkhori, Nourdine Bah, Stefan Boeing, Saeed Shoaie, Dimitrios Anastasiou

**Affiliations:** 1 Cancer Metabolism Laboratory, The Francis Crick Institute, 1 Midland Road, London NW1 1AT, UK; 2 Centre for Host-Microbiome Interactions, Faculty of Dentistry, Oral and Craniofacial Sciences, King’s College London, London SE1 9RT, UK; 3 Bioinformatics and Biostatistics Science Technology Platform, Francis Crick Institute, 1 Midland Road, London NW1 1AT, UK; 4 Science for Life Laboratory (SciLifeLab), KTH - Royal Institute of Technology, Tomtebodavägen 23, 171 65 Solna, Stockholm, Sweden

**Keywords:** Biological sciences, Physiology, Cellular physiology, Cancer

## Abstract

Dietary nutrient availability and gene expression, together, influence tissue metabolic activity. Here, we explore whether altering dietary nutrient composition in the context of mouse liver cancer suffices to overcome chronic gene expression changes that arise from tumorigenesis and western-style diet (WD). We construct a mouse genome-scale metabolic model and estimate metabolic fluxes in liver tumors and non-tumoral tissue after computationally varying the composition of input diet. This approach, called Systematic Diet Composition Swap (SyDiCoS), revealed that, compared to a control diet, WD increases production of glycerol and succinate irrespective of specific tissue gene expression patterns. Conversely, differences in fatty acid utilization pathways between tumor and non-tumor liver are amplified with WD by both dietary carbohydrates and lipids together. Our data suggest that combined dietary component modifications may be required to normalize the distinctive metabolic patterns that underlie selective targeting of tumor metabolism.

## Highlights

• A method to study effects of diet composition on mouse liver cancer metabolism
• Western diet increases glycerol and succinate production in both tumor and liver
• Western diet amplifies tumor/non-tumor tissue metabolic differences
• Dietary carbohydrates and fatty acids together promote tumor lipid metabolism

## Graphical abstract

**Figure:**
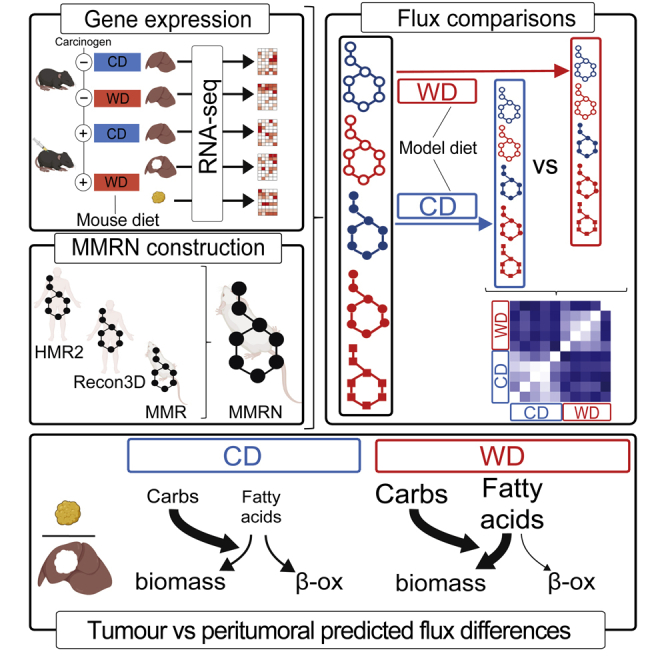

## Introduction

Cellular metabolic activities are largely determined by availability of nutrients and the expression of specific metabolic enzymes in cells.^1^^,^^2^ In multicellular organisms, metabolic enzyme expression in tissues is regulated by multiple mechanisms that operate at the cellular, tissue and organismal levels. Given that diet composition can have profound effects on body homeostasis and disease progression, understanding the interaction between dietary nutrient content and gene expression under conditions of clinical interest may offer insight into how diet modifications can be used to alter the course of disease. In this context, we examined the interplay between diet composition and gene expression in a mouse model of liver cancer.

A significant increase in the incidence of human liver cancer is projected over the next few decades with limited therapeutic options available.^3^^,^^4^^,^^5^ Similar to other tumor types, liver cancer can be promoted by “Western-style” diets (WD) that are rich in fat and processed sugars.^6^^,^^7^^,^^8^ Human and mouse studies indicate that chronic consumption of WD can lead to systemic dysregulation of insulin signaling and lipid metabolism; the ensuing inflammation and tissue damage are thought to promote mutations that lead to oncogenic transformation in hepatocytes.^7^^,^^8^^,^^9^

Both the nutrients in WD and systemic dysregulation of homeostatic mechanisms because of chronic WD consumption can influence, directly or indirectly, metabolic gene expression in hepatocytes.^10^^,^^11^^,^^12^ Concomitantly, oncogenic mutations impose metabolic wiring in hepatic tumors that is distinct from that of the surrounding host tissue and supports tumor growth and survival.^13^^,^^14^ However, it is unclear how the diet-induced chronic changes in gene expression in hepatic tissues influence the fueling of liver cancer metabolism by dietary nutrients. It is also unclear whether modulation of diet composition, alone, suffices to relieve the metabolic features that promote tumor growth.^15^^,^^16^^,^^17^ Investigation of such questions requires a global survey of metabolic activities in tissues because diverse dietary nutrients simultaneously fuel multiple metabolic pathways that are extensively interconnected. Despite significant progress in analytical methods and targeted tracing of metabolic activities *in vivo*, measurements of actual metabolic activities (fluxes) on a global scale remains challenging.^18^^,^^19^

Genome-scale metabolic models (GSMMs) are mathematical frameworks that comprehensively describe as many metabolic reactions, in a cell or organism, as possible, and can be used to predict metabolic fluxes at a whole-genome scale.^20^^,^^21^ To explore specific biological questions, metabolic input and intracellular fluxes in GSMMs can be constrained with information from various experimental data types.^22^ Metabolic input into GSMMs can be constrained with experimental measurements of metabolite consumption^23^^,^^24^^,^^25^ or, where such information is not readily obtainable, based on the content of the diet consumed by an animal.^26^ Computational methods can further be used to estimate enzyme activities from gene or protein expression datasets.^22^^,^^27^ Together, nutrient input and expression-based intracellular flux constraints significantly improve the accuracy of model predictions in specific biological contexts.^28^ Therefore, constraint-based modeling has been successfully used to simulate the effect of diet on metabolism^29^^,^^30^^,^^31^^,^^32^ or interrogate complex metabolic features in various diseases, including cancer, at a whole-genome scale.^23^^,^^33^^,^^34^^,^^35^^,^^36^^,^^37^^,^^38^ However, there have been no studies that systematically examine how various nutrient components in animal diet influence metabolic fluxes in GSMMs of mammalian tissues.

Several human GSMMs (hGSMMs) exist and include the Recon series (Recon1, 2 and 3D),^35^^,^^39^^,^^40^ the Human Metabolic Reaction series (HMR 1 and 2),^36^^,^^41^ and, more recently, Human-GEM.^34^ Individual hGSMMs have been previously used as templates to accelerate construction of GSMMs for other organisms of research interest, including the mouse.^26^^,^^42^^,^^43^^,^^44^ Given that the ensembles of metabolic reactions in existing hGSMMs do not fully overlap with each other, integration of multiple hGSMMs could be a useful way to generate more comprehensive mouse GSMM (mGSMM). However, such efforts are cumbersome, in part because of the use of nonstandard identifiers for components between existing models.^26^^,^^34^^,^^43^ New mGSMMs would be of great value given the wide-spread use of mice in metabolic research and, more broadly, as a pre-clinical model for human disease.

In this study, we construct a new mGSMM and use it to investigate how dietary nutrients and gene expression changes associated with chronic exposure to a WD combine to influence metabolism in the livers of mice with liver cancer.

## Results

### Changes in expression of genes that control metabolic processes by a tumor-promoting western-style diet

**Figure 1:**
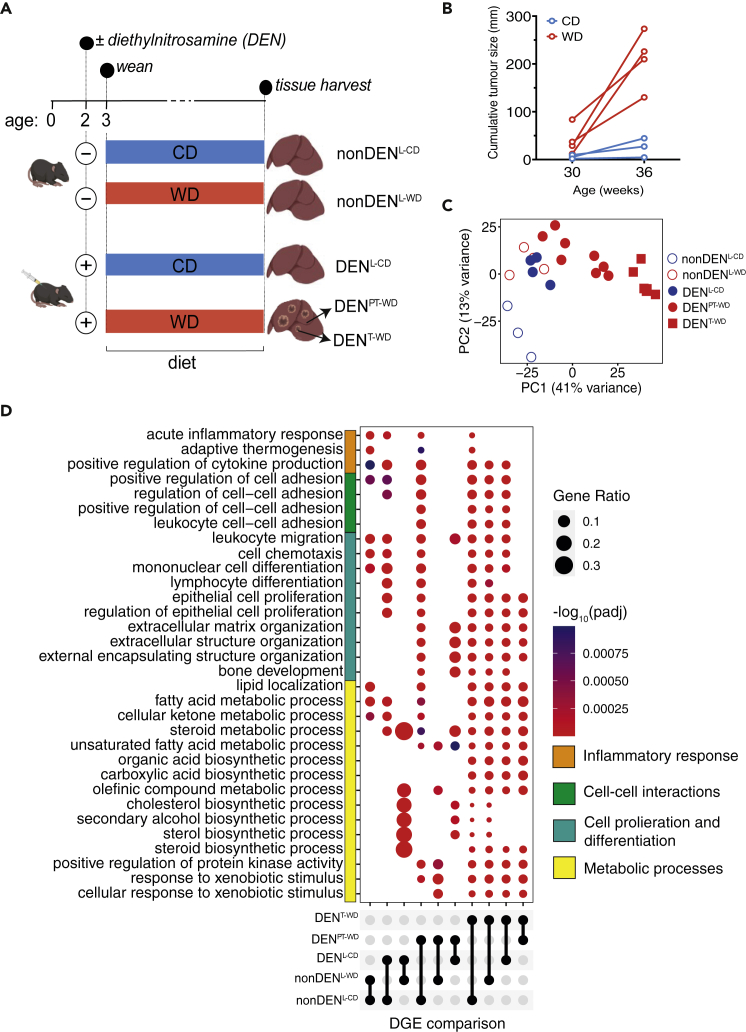
A western diet promotes mouse liver cancer development and elicits distinct hepatic gene expression profiles (A) Summary of the experimental mouse liver cancer model and tissue sampling times in this study. Mice at two weeks of age were injected with the carcinogen diethylnitrosamine (DEN). Starting at the time of weaning, mice were fed a western diet (WD), or a matched control diet (CD). At 36 weeks of age mice were culled and liver tissue was harvested for RNA-sequencing. (B) Cumulative tumor size of three DEN^CD^ mice and four DEN^WD^ mice at 30 and 36 weeks of age measured by magnetic resonance imaging (MRI). Tumor burden in DEN^WD^ mice increases significantly more over time than that in DEN^CD^ mice (paired t-test, p value <0.05). (C) Principal component analysis (PCA) of gene expression data derived from RNA-Sequencing analysis of tissue samples described in panel A. (D) Gene ontology (GO) biological process over-representation test for differentially expressed genes using the *enrichGO* function and visualised with the *dotplot* function from the *clusterProfiler* package. For each comparison, the bottom condition is used as baseline. Benjamini-Hochberg correction was used with a q-value cut-off of 0.01 and is represented by dot color. Dot size represents the fractional number of genes enriched within a particular biological process compared to the total gene set size.

To study the interplay between diet composition and diet-induced changes in gene expression in the context of hepatic tumor development, we injected mice with the carcinogen diethylnitrosamine (DEN), or left them untreated (nonDEN) and fed them either a western-style (WD) or control diet (CD)^7^ (henceforth, referred to as DEN^WD^/nonDEN^WD^ and DEN^CD^/nonDEN^CD^, respectively) (Figure 1A, Table S1).

Mice consumed either diet at similar rates (Figure S1A), however, more tumors were detectable by 25–29 weeks of age in DEN^WD^ compared to DEN^CD^ mice (Figure S1B). Between 30 and 36 weeks of age, tumor burden increased significantly in DEN^WD^ mice compared to DEN^CD^ mice (p = 0.02, paired t-test), and by 39 weeks 55% DEN^CD^ and 100% of DEN^WD^ mice had tumors (Figures 1B and S1B). These data confirmed the previously observed tumor-promoting effects of WD relative to CD.^7^ Tumors in DEN^CD^ mice were too small to reliably separate from peritumoral tissue, and because processes associated with aging may convolute comparison of heterochronous tumors, we did not attempt to further age DEN^CD^ mice to obtain resectable tumors.

To assess gene expression changes caused by diet, carcinogen and tumor development, we analyzed the transcriptional profiles of available tumors and liver tissues using RNA sequencing. Principal component analysis (PCA) revealed two major PCs: PC1 (accounting for 41% of variance) was associated with effects of DEN and PC2 (13%) with diet (Figure 1C). Gene ontology (GO) analysis of differentially expressed genes across all pairwise comparisons revealed an enrichment in processes related to (a) inflammation, which were broadly linked to both diet and DEN; (b) cellular proliferation and cell-cell interactions, primarily associated with tumor or peritumoral tissues, and (c) metabolic processes, several of which were related to lipid metabolism and emerged, at varying degrees, as a function of diet, DEN or the transformation state of the tissue (Figure 1D).

These observations suggest that chronic exposure to WD is associated with changes in expression of genes that mediate metabolic processes which, in addition to dietary nutrient availability, could influence both the rate and functions of tissue metabolism.

### Construction of MMRN, a new mouse genome-scale metabolic network

**Figure 2:**
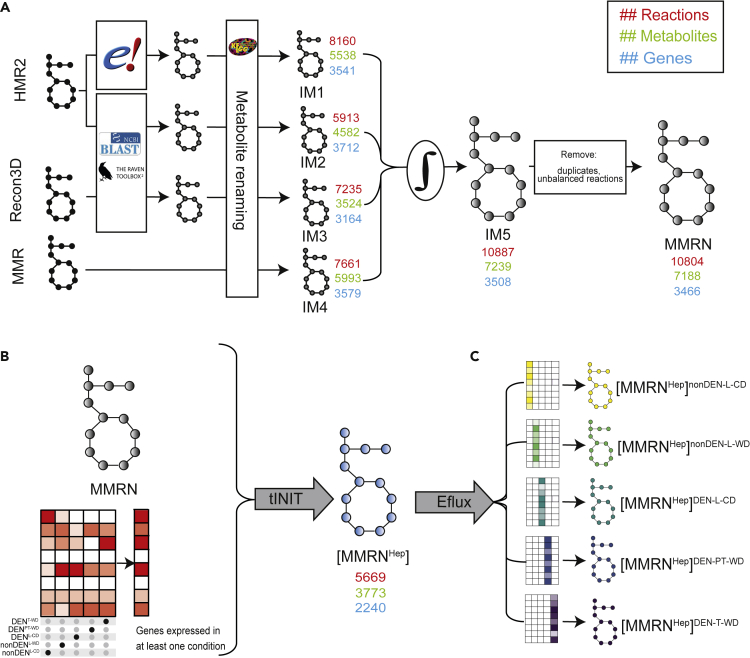
Outline of strategy for the construction of MMRN and derivative context-specific hepatic tissue models (A) Construction of Mouse Metabolic Reaction Network (MMRN) from four intermediate networks (IM1-4). For IM1, proteins in HMR2 were replaced with their known mouse orthologs from the Ensembl database. The RAVEN 2.0 Toolbox was used to generate IM2 and IM3 based on the sequence homology of mouse proteins to the protein sequences encoded by genes in HMR2 and Recon3D, respectively. Metabolites in IM1-3 were renamed to their corresponding KEGG identifiers (Table S2); similarly renaming metabolites in MMR resulted in IM4. IM1-4 were integrated into a single network, IM5. Duplicate and elementally unbalanced reactions were removed from IM5 to obtain MMRN. The effect of each step on key model attributes is shown by the number of reactions (red), metabolites (green) and genes (blue) adjacent to each model. (B) RNA-sequencing data were used to identify all genes that were expressed in at least one of the experimental conditions shown in Figure 1A. This list of genes, together with growth tasks and MMRN were used as input for the tINIT algorithm^45^ to reconstruct a generic hepatic GSMM, [MMRN^Hep^]. MMRN and [MMRN^Hep^] in MATLAB, SMBL and Excel format with corresponding code for simulations are available at https://github.com/sysbiomelab/MMRNHep. (C) [MMRN^Hep^] was further constrained using an adapted Eflux method (see STAR Methods) and gene expression data for each experimental condition to produce five condition-specific GSMMs (csGSMMs). The Eflux method^46^ imposes flux boundaries on individual reactions based on TPM expression of all genes predicted to catalyze each reaction.

With the outlook of assessing how the observed gene expression changes induced by diet may modulate the metabolism of dietary nutrients across tissues, we generated an updated mGSMM. To this end, we constructed, then combined four intermediate models (IMs) based on existing human and mouse GSMMs (Figure 2A).

Specifically, we directly replaced proteins in HMR2 with their known mouse orthologs to yield IM1. We also reconstructed two metabolic models based on the sequence homology of mouse proteins to the protein sequences encoded by genes in HMR2 (IM2) and Recon3D (IM3), respectively. We finally considered MMR, the most advanced mouse GSMM at the time we started our study, as IM4. Only 60.5% of genes and 41.9% of metabolites overlapped between IM1-4 (Figure S2A), indicating significant non-redundancy. Integration of IM1-4 resulted in a new network, IM5, from which we identified and removed 83 reactions that were elementally imbalanced (see STAR Methods). The resulting final network, which we named Mouse Metabolic Reaction Network (MMRN), could perform 56 common metabolic tasks,^45^ including the production of biomass. Assessment of MMRN using the MEMOTE framework^47^ revealed a significant improvement compared to the source models used for our reconstruction (Table S3). Moreover, MMRN MEMOTE scores are comparable to several of the individual model attribute scores for Mouse1,^44^ the most recent mGSMM published during the course of our work, with the exception of stoichiometric consistency. Further exploration revealed that this low score is attributable to reactions involved in lipid metabolism (see STAR Methods). On the other hand, MMRN has fewer orphan reactions than Mouse1 and no carbon-imbalanced reactions compared to any of the other mGSMMs (Table S4, Figure S2B). MMRN is available at https://github.com/sysbiomelab/MMRNHep.

### csGSMMs selectively take up nutrients for biomass production

We next constrained MMRN with all the genes that we found expressed in at least one condition of our collective gene expression dataset to generate a hepatic GSMM, [MMRN^Hep^] (Figure 2B). We further constrained [MMRN^Hep^] with gene expression from different experimental conditions and tissues, as well as O_2_ consumption and CO_2_ production rates of mice measured in metabolic cages (Table S5, STAR Methods) to generate five context-specific GSMMs (csGSMMs) (Figure 2C). We then determined how CD or WD alter flux distributions in these csGSMMs, using biomass production as the objective function (Tables S6 and S7). Henceforth we use the notation ${\left[{MMRN}^{Hep}\right]}_{Y}^{X}$, where X indicates the gene expression constraint (nil when no gene expression constraint is applied) and Y is the diet given to the model.

**Figure 3:**
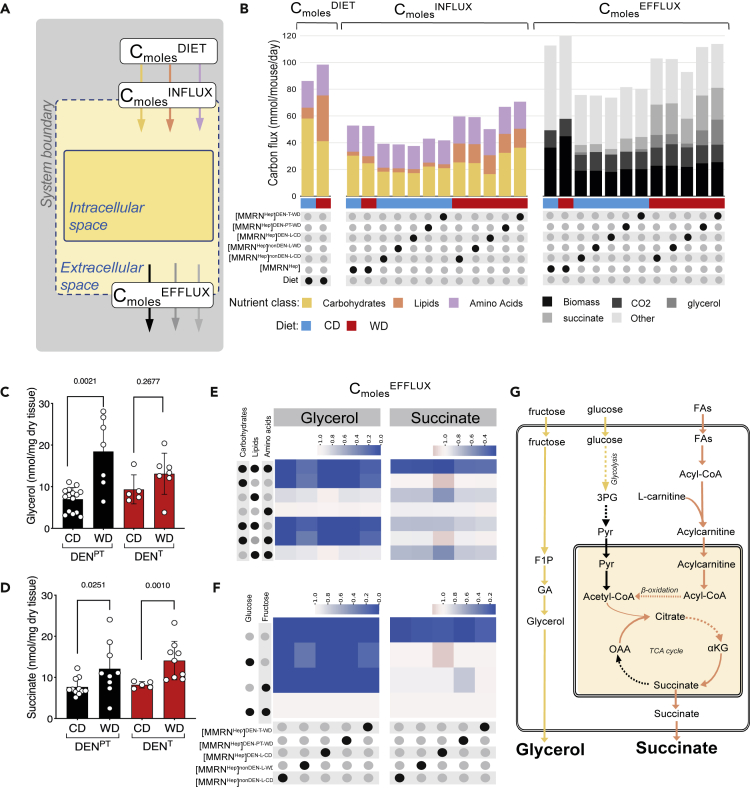
Selective dietary nutrient uptake and increased production of glycerol and succinate elicited by WD across all context-specific hepatic models in this study (A) Schematic illustrating relation between input and output carbon fluxes in the context of flux balance analysis (FBA) experiments shown in panels B, E, F and Figure S3. C_moles_^DIET^ represents the carbon flux for each metabolite that is available from the diet to [MMRN^Hep^] and was calculated based on the known diet composition and daily diet consumption per mouse (Tables S1and S6 and Figure S1A). The dashed line represents a computational pseudo-boundary set to allow influx of metabolites from the diet into the extracellular space of the model. For a given metabolite, C_moles_^INFLUX^ and C_moles_^EFFLUX^ denote the flux of carbons of this metabolite taken up or produced, respectively, by [MMRN^Hep^]. (B) C_moles_^DIET^ values of each of the major dietary nutrient classes (carbohydrates, lipids and amino acids) in CD and WD. Corresponding values of individual nutrient components is shown in Figure S3A. C_moles_^INFLUX^ and C_moles_^EFFLUX^ for [MMRN^Hep^] and csGSMMs models provided with either CD or WD. (C) Amounts of glycerol in DEN^PT^ and DEN^T^ tissues from mice fed WD compared to respective tissues from CD-fed mice measured by GC-MS. Data are represented as mean ± SD. Numbers above bars indicate p values determined by a two-tailed Mann-Whitney test (n = 5–15 mice). (D) Amounts of succinate in DEN^PT^ and DEN^T^ tissues from mice fed WD compared to respective tissues from CD-fed mice measured by GC-MS. Data are represented as mean ± SD. Numbers above bars indicate p values determined by a two-tailed Mann-Whitney test (n = 5–15 mice). (E) Systematic Diet Component Swap (SyDiCoS) to assess the role of WD components on glycerol and succinate production flux in csGSMMs. C_moles_^DIET^ values for all three major diet component classes (carbohydrates, lipids and amino acids) in the WD were swapped individually or in combination with the corresponding C_moles_^DIET^ values in CD while leaving the remaining dietary C_moles_^DIET^ values of the WD unaltered. The swapped component(s) are indicated by black dots on the left. The color scale represents the ratio of glycerol production flux or succinate production flux in models provided with the swapped diet relative to the respective fluxes in models provided with WD, calculated for each csGSMM shown at the bottom of panel (F). (F) Assessment of the role of glucose or fructose from WD on glycerol and succinate production. csGSMMs shown at the bottom were provided WD containing only glucose or fructose (using their respective C_moles_^DIET^ values found in WD) as indicated by the black dots on the left, or both sugars (equivalent to the original WD composition) while leaving the C_moles_^DIET^ values for lipids and amino acids in WD unaltered. The color scale represents the ratio of glycerol production flux or succinate production flux in models provided with the modified WD inputs relative to the respective fluxes in models using the original WD composition, calculated for each csGSMM shown at the bottom. (G) Metabolic pathways that lead to increased production of glycerol and succinate in WD from fructose and FAs, respectively, derived from inspection of the flux distributions of csGMMS under various SyDiCoS conditions (panels E, F and Table S7). FAs: fatty acids; 3 PG: 3-phosphyglycerate; Pyr: pyruvate; F1P: fructose 1-phosphate; GA: glyceraldehyde; αKG: α-ketoglutarate; OAA: oxaloacetate.

The total flux of carbons (C_moles_^DIET^, Figure 3A) that were available from the WD was higher than that for CD (Figure 3B). Accordingly, the flux of carbons taken up (C_moles_^INFLUX^, Figure 3A) by WD-fed csGSMMs was higher than that of CD-fed csGSMMs, and higher than [MMRN^Hep^] fed with either diet (Figure 3B). However, we found no difference in C_moles_^INFLUX^ between ${\left[{MMRN}^{Hep}\right]}_{WD}$ and ${\left[{MMRN}^{Hep}\right]}_{CD}$. Furthermore, C_moles_^INFLUX^ values were lower than C_moles_^DIET^ for both diets in all models, and the relative C_moles_^INFLUX^ of individual diet components did not reflect the corresponding C_moles_^DIET^ values for those nutrients (Figure S3A). Together, these observations suggest that [MMRN^Hep^] limits the amounts and types of nutrients it takes from the diet for optimal production of biomass, irrespective of condition-specific gene expression; they further indicate that gene expression together with dietary composition, rather than nutrient availability alone, dictate nutrient uptake by our models.

Consistent with a carbon-balanced model, increased C_moles_^INFLUX^ in WD compared to CD was mirrored by higher total C_moles_^EFFLUX^ values for all models (Figure S3B), which was mostly accounted for by higher production of glycerol and succinate (Figures 3B and S3B). We confirmed this prediction by metabolomics analyses of tissues, which revealed increased levels of glycerol and succinate in both DEN^T^ and DEN^PT^ tissues (Figures 3C and 3D).

To determine whether particular dietary nutrient classes influenced C_moles_^EFFLUX^, we swapped the amount of each dietary component class in WD with the respective amount of that component in CD while keeping the remaining WD composition unchanged (henceforth, WD^X(CD)^ refers to diets where X = WD diet component that was swapped to its respective CD value) (Figure 3E). We collectively refer to any computational manipulation of diet composition as Systematic Diet Composition Swap (SyDiCoS). csGSMMs in WD^lipid(CD)^ revealed a dependence of succinate production on the increased lipid content of WD, which drove higher TCA cycle flux. The quantitative increase in glycerol production by WD was abrogated with WD^carbs(CD)^. Further investigation of the flux distributions with WD^carbs(CD)^ (Table S7) showed that WD-derived fructose accounted for increased glycerol production (Figures 3F and 3G). However, when used as the sole dietary sugar, fructose was diverted to sustain glycolysis, and glycerol production ceased (Table S7).

### Gene expression and dietary nutrient availability dictate differential fate of FAs in tumors and peritumoral liver

Excess glycerol and succinate produced in WD-fed tissues could influence tissue physiology in a non-cell autonomous manner, through mechanisms that cannot be captured by our single-tissue models.^48^^,^^49^^,^^50^ We therefore focused further investigations on the effects of diets on biomass.

**Figure 4:**
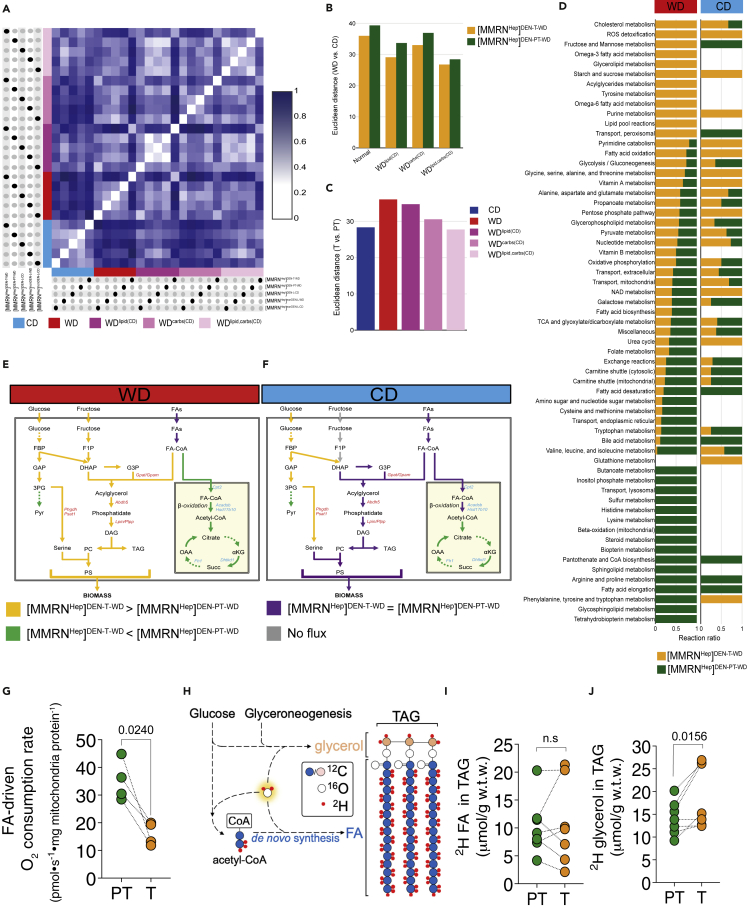
Gene expression together with dietary nutrient availability dictate differential fate of FAs in tumors and peritumoral liver (A) Effects of diet composition on flux distribution differences between csGSMMs assessed by SyDiCoS. FBA was used to calculate the flux distribution for each csGSMM provided with CD, WD, WD^lipid(CD)^, WD^carbs(CD)^ and WD^lipid,carbs(CD)^. The color scale represents the Euclidean distance values calculated in a pairwise manner between each of the flux distributions and plotted relative to the maximum distance value across all comparisons. (B) Relative response to changes in diet composition of the flux distributions of tumoral or peritumoral models. Absolute Euclidean distances (from panel A) for either [MMRN^Hep^]^DEN^^−^^T^^−^^WD^ or [MMRN^Hep^]^DEN−PT^^−^^WD^ under different SyDiCoS conditions are plotted. (C) Effect of changes in diet composition on the flux distributions differences between tumoral and peritumoral models. Absolute Euclidean distances (from panel A) between [MMRN^Hep^]^DEN−T^^−^^WD^ or [MMRN^Hep^]^DEN−PT^^−^^WD^ under different SyDiCoS conditions are plotted. (D) Subsystems that include at least one reaction that carries flux in [MMRN^Hep^]^DEN−T^^−^^WD^ or [MMRN^Hep^]^DEN−PT^^−^^WD^ on either WD or CD. In each of these subsystems, the proportion of reactions with higher flux in [MMRN^Hep^]^DEN−T^^−^^WD^ compared to [MMRN^Hep^]^DEN−PT^^−^^WD^ on either WD or CD is plotted. For each diet, a reaction ratio = 1 for [MMRN^Hep^]^DEN−T^^−^^WD^ in a given subsystem indicates that all reactions in that subsystem have higher flux compared to [MMRN^Hep^]^DEN−PT^^−^^WD^. (E and F) Flux differences between tumoral and peritumoral models fed either WD (E) or CD (F). These two networks are schematic representations of the metabolic network shown in Data S1, which comprises all the reactions of subsystems from panel (D) that have a reaction ratio = 1 (either all reactions that carry higher flux in [MMRN^Hep^]^DEN−T^^−^^WD^ or in [MMRN^Hep^]^DEN−PT^^−^^WD^) and partake in lipid and carbohydrate metabolism. Differential fluxes for T and PT are colored according to the legend at the bottom of these panels. FBP: fructose 1,6-bisphosphate; GAP: glyceraldehyde 3-phosphate; 3 PG: 3-phosphoglycerate; Pyr: pyruvate; F1P: fructose 1-phosphate; DHAP: dihydroxyacetone phosphate; G3P: glycerol 3-phosphate; DAG: diacylglycerol; TAG: triacylglycerol; PC: phosphatidylcholine; PS: phosphatidylserine; FAs: fatty acids; FA-CoA: fatty acyl-CoA; αKG: α-ketoglutarate; Succ: succinate; OAA: oxaloacetate; *Phgdh*: Phosphoglycerate dehydrogenase; *Psat1*: Phosphoserine aminotransferase 1; *Gpat*: Glycerol 3-phosphate acyltransferase; *Gpam*: Glycerol 3-phosphate acyltransferase 1, mitochondrial; *Abdh5*: 1-acylglycerol 3-phosphate O-acyltransferase; Lpin: Phosphatidate phosphatase; *Plpp*: Pyridoxal phosphate phosphatase; *Mogat1*: Monoacylglycerol O-acyltransferase 1; *Cpt2*: Carnitine palmitoyltransferase 2; *Acadsb*: Acyl-CoA dehydrogenase short/branched chain; Hsd17b10: Hydroxysteroid 17-β dehydrogenase 10; *Dhtkd1*: Dehydrogenase E1 and transketolase domain containing 1; *Fh1*: Fumarate hydratase 1. (G) Comparison of FA-driven oxygen consumption rates in mitochondria isolated from liver tumors (T) or peritumoral (PT) tissues of mice treated as described in Figure 1A. Statistical significance determined by two-tailed paired t-test (n = 4 different mice, each providing a paired T and PT tissue sample from which mitochondria were isolated; oxygen consumption was measured in parallel for each T/PT sample pair). (H) Schematic showing metabolic routes of ^2^H incorporation into the glycerol backbone and fatty-acyl chains in a triglyceride (TAG) molecule after administration of ^2^H_2_O to mice. (I) Measurement of *de novo* synthesized fatty-acids (as outlined in H) in TAGs extracted from tumor (T) and peritumoural (PT) tissues. (J) Measurement of *de novo* synthesized glycerol (as outlined in H) in TAGs extracted from tumor (T) and peritumoral (PT) tissues. Statistical significance in (I) and (J) determined by Wilcoxon matched-pairs signed rank test (n = 7 different mice, each providing a paired T and PT tissue sample).

WD led to a modest increase of biomass production in all csGSMMs compared to the respective CD-fed GSMMs (Figure 3B), demonstrating that, whereas our models are robust and withstand a massive overhaul of diet composition, they are also sensitive enough to detect the resulting flux changes. However, the flux distributions of all csGSMMs differed from each other, with ${\left[{MMRN}^{Hep}\right]}_{CD}^{DEN-T-WD}$ being the most distinct. Furthermore, although WD shifted the flux distributions of all models, it amplified the differences between the flux distributions of the tumor and that of non-tumor models (Figure 4A, Table S8). These observations indicated that similar increases in biomass production in tumor and non-tumor tissues induced by WD are associated with distinct metabolic pathway activities. Henceforth, to simplify further exploration of such pathways, we focused on the comparisons between ${\left[{MMRN}^{Hep}\right]}^{DEN-T-WD}$ and ${\left[{\text{MMRN}}^{\text{Hep}}\right]}^{DEN-PT-WD}$.

WD caused a greater shift, relative to CD, in the flux distributions of ${\left[{MMRN}^{Hep}\right]}^{DEN-T-WD}$ than those of the ${\left[{\text{MMRN}}^{\text{Hep}}\right]}^{DEN-PT-WD}$ indicating that the tumor metabolic network is primed for a greater response to WD than the peritumoral model (Figures 4A and 4B). Notably, the WD-induced increase in tumor/non-tumor divergence was completely reversed only when both lipids and carbohydrates in WD were replaced with their respective CD content (Figures 4A and 4C).

To explore specific pathways that underlie differential response of tumor and peritumoral tissue models to WD driven by carbohydrates and lipids, we first identified subsystems of all reactions that carried flux in ${\left[{MMRN}^{Hep}\right]}_{WD}^{DEN-T-WD}$ (henceforth “T”) and ${\left[{MMRN}^{Hep}\right]}_{WD}^{DEN-PT-WD}$ (henceforth “PT”). Further analysis revealed those subsystems that were differentially engaged in the tumor and peritumoral tissue models; among them were several subsystems involved in lipid and carbohydrate metabolism (Figure 4D). We then constructed a network comprising all the reactions in the subsystems related to lipid and carbohydrate metabolism that showed differential flux exclusively in the T or PT in WD but not in CD (Data S1). From this network, we extracted a single fully connected subnetwork that is connected to biomass production, and calculated flux differences between ${\left[{MMRN}^{Hep}\right]}_{WD}^{DEN-T-WD}$ and ${\left[{MMRN}^{Hep}\right]}_{WD}^{DEN-PT-WD}$ (Figure 4E, Table S9), and between ${\left[{MMRN}^{Hep}\right]}_{CD}^{DEN-T-WD}$ and ${\left[{MMRN}^{Hep}\right]}_{CD}^{DEN-PT-WD}$ (Figure 4F, Table S9).

Comparison of the reaction fluxes in this network revealed a distinct diet-dependent fate of dietary FA’s in the tumor and the peritumoral tissue models. On one hand, FA uptake and FA-derived acyl-CoA synthesis fluxes were higher in WD than in CD for both the T and PT models, and equal between T and PT models in each diet. However, our models predicted increased use of FA-CoA for lipid macromolecule synthesis and decreased use for β-oxidation through the mitochondrial carnitine shuttle in ${\left[{MMRN}^{Hep}\right]}_{WD}^{DEN-T-WD}$ compared to ${\left[{MMRN}^{Hep}\right]}_{WD}^{DEN-PT-WD}$. These flux differences correlated with higher expression of lipid synthesis genes and lower expression of mitochondrial lipid transport and β-oxidation genes in tumors (Figure S4A, Table S10). Consistent with this observation, mitochondria purified from tumor tissue respired significantly less on FAs than mitochondria purified from peritumoral tissue (Figure 4G).

Notably, both ${\left[{MMRN}^{Hep}\right]}_{CD}^{DEN-T-WD}$ and ${\left[{MMRN}^{Hep}\right]}_{CD}^{DEN-PT-WD}$ used FA-derived acyl-CoA equally for either biomass or β-oxidation, whereas differential fate of acyl-CoA between ${\left[{MMRN}^{Hep}\right]}^{DEN-T-WD}$ and ${\left[{\text{MMRN}}^{\text{Hep}}\right]}^{DEN-PT-WD}$ persisted when these models were fed either WD^lipids(CD)^ or WD^carbs(CD)^ (Figures S4B and S4C). This observation suggested that differential expression of β-oxidation and lipid synthesis genes does not suffice to drive divergence of FA metabolic fates in the absence of altered dietary composition of both FAs and carbohydrates.

Our flux predictions showed increased fluxes of glycolysis and fructolysis, which contribute precursors for lipid synthesis, in ${\left[{MMRN}^{Hep}\right]}_{WD}^{DEN-T-WD}$ compared to ${\left[{MMRN}^{Hep}\right]}_{WD}^{DEN-PT-WD}$. Increased serine synthesis from carbohydrates persisted in ${\left[{MMRN}^{Hep}\right]}_{CD}^{DEN-T-WD}$ compared to ${\left[{MMRN}^{Hep}\right]}_{CD}^{DEN-PT-WD}$, indicating no impediment in serine synthesis that would explain attenuated lipid synthesis in CD. In contrast, incorporation of glycerol 3-phosphate (G3P) through glycerol 3-phosphate acyltransferase (encoded by *Gpat*/*Gpam*) into lipids ceased for both models in CD but was increased in ${\left[{MMRN}^{Hep}\right]}_{WD}^{DEN-T-WD}$ compared to ${\left[{MMRN}^{Hep}\right]}_{WD}^{DEN-PT-WD}$. In agreement with a role for G3P-fuelled esterification in driving increased lipid synthesis in T vs PT, we found that, whereas both tumor and peritumoral mouse tissues synthesized FA’s at comparable rates *in vivo* (Figures 4H and 4I), tumors showed increased incorporation of newly synthesized glycerol into lipids (Figures 4H and 4J).

In summary, exploration of flux distributions in our new mouse csGSMM models revealed a convergence of nutrient availability and gene expression in driving a differential response of tumors and peritumoral tissue to alterations in diet composition.

## Discussion

In liver cancer that develops in the background of chronic WD feeding, both the host liver and the emerging tumors have distinct metabolic gene expression from each other and compared to healthy livers. Such differential gene expression can impact the ability of dietary modulations to reverse metabolic activities that underlie liver pathologies. It is unclear whether diet modulation is sufficient to overcome metabolic wiring that occurs in the background of chronic gene expression changes.

In this study we develop a roadmap toward exploring this question in a mouse model of liver cancer. We construct a new mGSMM, MMRN, and use gene expression data from liver tissues to generate csGSMMs. We then explore changes in the flux distributions of these csGSMMs in response to varying diet compositions through a new approach called Systematic Diet Composition Swap (SyDiCoS). These investigations provide evidence for a combined effect of nutrient availability and gene expression in determining the characteristic metabolic profiles of tumors and response to diet modulation.

### Integration of multiple existing GSMMs aids reconstruction of an expanded mGSMM

Bottom-up approaches to reconstruct GSMMs are labour-intensive and time consuming.^51^ The availability of whole-genome sequencing data has accelerated the reconstruction of GSMMs for model organisms by replacing genes in a template hGSMM with known mouse orthologues.^44^ However, in this approach, metabolic reactions catalyzed by enzymes without an ortholog in the target organism will not be represented in the final reconstruction.^52^^,^^53^ Furthermore, different hGSMM series comprise non-redundant metabolite and gene components, so, derivative mGSMMs that use either one or the other as a template, may be incomplete. Given the extensive interconnectivity of mammalian metabolic pathways and that the functions of a significant fraction of the mammalian metabolic network remains poorly explored, maximal coverage is desired to improve the ability of GSMMs to model metabolism holistically.

To overcome these limitations, we used both HMR2 and Recon3D, and complemented classic gene orthology-based gene replacement methods with protein sequence homology-based identification of mouse orthologous genes. Used together with a previous mGSMM, MMR, this approach yielded MMRN. Compared to other mouse models at the time we initiated our study, MMRN has expanded coverage of the mouse metabolic network and overall improved attributes evaluated by MEMOTE, a framework of standardized tests that aims at consistently assessing GSMMs.^47^ Notably, although the MEMOTE performance of MMRN also compares well to that of Mouse1 (the latest published mGSMM), MMRN scored less for stoichiometric consistency. This was exclusively attributable to reactions involving lipid metabolites, which highlights a well-known challenge in GSMM reconstruction that arises from the difficulties in defining the exact molecular composition of such metabolites and, by extension, their exact stoichiometries in reactions that interconvert them.^34^^,^^51^^,^^54^ Although construction of new GSMMs based on pre-existing ones has limitations,^55^ the fact that IMs from different hGSMMs and from protein homology-based methods show significant non-redundancy demonstrates that integration of pre-existing models and improvement of orthology information can facilitate reconstruction of model organism GSMMs based on existing hGSMMs.

### SyDiCoS aids interrogation of GSMMs functions

Application of GSMMs to specific biological questions is commonly aided by constraining metabolic fluxes with context-specific data.^22^^,^^31^ Various environmental and cell-intrinsic factors can alter gene expression to change enzyme abundance and, by extension, metabolic fluxes. Therefore, substantial effort has been invested in developing methods to constrain intracellular fluxes using gene expression data.^22^^,^^28^ On the other hand, availability of nutrients can also vary under different dietary regimes, physiological conditions, and between different tissue environments. The impact of nutrient availability has been previously studied in microbial GSMMs^56^^,^^57^^,^^58^^,^^59^ and cultured cancer cells.^23^^,^^24^ Information on diet composition has also been previously used to constrain mammalian tissue GSMMs.^31^^,^^32^ However, to the best of our knowledge, there are no studies that systematically assess how the functions of a given mammalian tissue csGSMM are modulated by the availability of individual nutrient classes, alone or together. To this end, for our study we developed SyDiCoS, where we compare flux distributions between csGSMM that we provide with diets in which we have modified all or select nutrient components.

A complete DiCoS from WD to CD as input for the models revealed that all csGSMMs took up more nutrients from WD than from CD. WD has more FA-derived and fewer carbohydrate-derived carbons, and a higher overall carbon content per unit weight of consumed diet. However, neither the total amount nor the amounts of specific nutrient classes taken up by csGSMMs reflected the amounts of these nutrients found in the input diet. For example, all csGSMMs consumed more carbohydrates in WD than CD, although the amount of carbohydrates in WD is lower than that of CD (Figure 3B).

Consistent with carbon-balanced models, we found increased efflux of products in WD-fed than CD-fed csGSMMs, which SyDiCoS revealed to be largely due to increased glycerol and succinate efflux. In a variant of SyDiCoS, we selectively swapped specific WD components and found that increased glycerol efflux is derived from carbohydrates whereas increased succinate efflux is attributable to lipid catabolism. We speculate that if these metabolites efflux out of cells as our models predict, they may mediate metabolic activities between cells or tissues. For example, glycerol is a precursor for carbohydrate and lipid synthesis in the normal liver and its metabolism is perturbed in metabolic disease.^60^^,^^61^^,^^62^^,^^63^ Furthermore, recent evidence suggests that succinate may have immunomodulatory roles within the liver and is increased in the blood of obese individuals, although the exact source of this succinate is under debate.^48^^,^^64^

Together, the application of SyDiCoS in various csGSMMs revealed that, irrespective of differences in gene expression, all csGSMMs limit both how much and which nutrients they take up from the diet, and what they produce to fulfill a given objective function.

### Combined effects of diet and gene expression underlie differences between tumor and peritumoral tissue metabolism

A complete WD-to-CD DiCoS caused a significant shift in the flux distributions of all csGSMMs. On the other hand, we also detected differences in flux distributions between csGSMMs on each diet, with the tumors being the most distinct. WD-to-CD DiCoS amplified these flux distribution differences. We therefore focused on comparisons between the flux distributions of tumor and peri-tumoral models because such differences are of both diagnostic as well as therapeutic interest. Identification of the pathways that account for flux differences is challenging as it requires either sophisticated network analysis algorithms or laborious manual exploration of the flux distributions.^28^^,^^65^ Selective DiCoS for specific WD components allowed us to attribute the amplification of flux distribution differences between T and PT to a combined effect of both dietary FAs and carbohydrates, and to generate a minimal subnetwork that focuses on the relevant pathways and is amenable to further interrogation.

With WD, both tumor and PT models took up FAs at similar rates. However, increased expression of genes that mediate lipid anabolic pathways and decreased expression of genes in β-oxidation resulted in a higher use of these FAs for biomass production and away from mitochondrial oxidation in T compared to PT models. Our models also predict increased production of precursors for lipid biomass derived from carbohydrates through increased expression of genes in two pathways. On one hand, increased expression of serine synthesis genes provides serine for phosphatidyl-serine synthesis; on the other hand, fructose breakdown produces DHAP and G3P that are used as backbone for acylglycerol production, through a series of reactions encoded by *Gpat*, *Abdh5*, *Lpin* and *Mogat*. SyDiCoS revealed that these lipid gene expression patterns sufficed to drive differential lipid metabolism between T and PT when either lipids or carbohydrates were normalized to CD levels, however, in a full swap to CD, this differential metabolism ceased. In contrast, increased serine synthesis in T relative to PT persisted in all diet inputs tested but in the absence of lipids serine did not contribute to lipid biomass more in T than in PT. Even though higher glucose uptake and flux to DHAP persisted in T with CD, it was not sufficient, alone, to sustain acylglycerol synthesis, even with increased *Gpat* expression, likely because fructose uptake ceased in CD.

Several of the pathways and nutrients we describe here have been shown to promote liver disease and cancer.^66^^,^^67^^,^^68^ Inhibition of pathways that provide serine attenuates myc-driven tumor growth, and high fructose consumption has been implicated in obesity and liver disease.^69^^,^^70^^,^^71^^,^^72^ Based on this type of evidence, diet modulation is increasingly pursued, both in the lab and in the clinic, as a means to reverse liver pathologies and attenuate cancer progression. Whether reverting to healthier diets suffices to normalize the metabolic activities driven by gene expression changes associated with chronic pathology is not clear. Even if this were the case, it is also uncertain that modulation of tissue metabolism can overcome the systemic signals and inflammation that often underlie liver diseases. In a scenario that tests whether a switch to a healthier diet might affect the course of the disease, our SyDiCoS approach aims at addressing the specific influence of nutrients on metabolism and needs to be integrated into the broader understanding of systemic effects of diet. In such a context, our observations would suggest that selectively altering diet composition may only mitigate particular metabolic branches of tumor metabolism and that inhibition of others may require combined modulation of multiple dietary substrates.

### Limitations of the study

MMRN contains reactions, several of which involved in lipid metabolism, with stoichiometric inconsistencies (STAR Methods-benchmarking of MMRN) that may underlie the relatively low mass balance MEMOTE score of MMRN (Table S3). This reflects a well-known challenge in GSMM reconstruction because of the fact that the exact mass and stoichiometry of reactions in lipid metabolism are hard to define from existing biological knowledge. Historically, in GSMM reconstructions, lipid fatty acid chains have been generically represented as R-groups and treated differently across lipid species and different models.^54^^,^^73^ More recently, one way to enforce stoichiometric consistency and mass balance for lipid metabolites has been to ensure consistent usage of R-groups across different reactions.^34^ Despite such extensive manual curation,^51^ and although this approach ensures computational consistency, it does not correct unknown formulas for these metabolites in a way that faithfully represents real lipid macromolecule composition in tissues (which, currently, is very hard to define and can vary between tissues). Although we experimentally validated the key predictions of our models, future improvements in GSMM reconstruction that address this limitation may assist in broadening the predictive capacity and accuracy of GSMMs, including MMRN.

The cellular composition of both the liver and tumors is heterogeneous and can change as a function of time, location within the tissue, and hormonal signals.^74^ Hepatocytes comprise approx. 60% of hepatic cell number but can contribute as much as 90% to hepatic biomass.^75^^,^^76^ Therefore, flux distributions of GSMM constrained with bulk RNA-Seq data would be predicted to predominantly reflect metabolism in hepatocytes and cancer cells, in the respective models. Nevertheless, hepatocytes themselves are metabolically heterogeneous, in part because of developmental transcriptional programmes.^74^ Single-cell data combined with approaches similar to those used to model metabolism of microbial communities^59^^,^^77^^,^^78^ may prove useful for elucidating the relevance of intercellular metabolic interactions and heterogeneity in determining tissue functions.^79^

The use of mouse diet composition as input to csGSMMs also has limitations. Although some dietary nutrients may reach the liver intact in some physiological settings, others can be digested in the gut lumen or metabolized by the microbiome before they reach tissues through the circulation.^80^^,^^81^^,^^82^ Also, even when information on circulating metabolite concentrations is available, it cannot be readily used to constrain uptake fluxes. Currently, there is no consistent framework for estimating which nutrients are available to tissues given a specific diet composition or blood metabolite concentrations; similar to our approach, several studies use the absolute dietary content as input to mammalian tissue models.^26^^,^^83^ The development of new methods to accurately model tissue-specific input would improve the performance of tissue-specific models. The availability of robust GSMMs and SyDiCoS, or similar approaches, to systematically interrogate the contributions of dietary components, alone and in combination, will likely be essential for developing rational dietary interventions.

## STAR★Methods

### Key resources table

**Table:** 

REAGENT or RESOURCE	SOURCE	IDENTIFIER
**Chemicals, peptides, and recombinant proteins**		

Diethylnitrosamine (DEN)	Merck	Cat# N0258
Control diet (CD)	TestDiet	AIN-93G
Western diet (WD)	TestDiet	AIN-76A
^2^H_2_O	Merck	Cat# 151882
ADP	Merck	Cat# A4386
Octanoyl-carnitine	TOCRIS Bioscience	Cat# 0605

**Critical commercial assays**		

ISOLUTE® NH2 SPE columns	Biotage, UK	Cat# 470-0010-A

**Deposited data**		

RNA-sequencing data used in this study	This paper	GEO: GSE199899
Original code generated in this study	This paper	Zenodo: https://doi.org/10.5281/zenodo.7520583

**Experimental models: Organisms/strains**		

*Mus musculus* C57BL/6J	The Jackson Laboratory	RRID:IMSR_JAX:000664

**Software and algorithms**		

RSEM	Li and Dewey^84^	http://deweylab.github.io/RSEM/
STAR	Dobin et al.^85^	https://github.com/alexdobin/STAR
RSeQC	Wang et al.^86^	https://rseqc.sourceforge.net/
RNA-SeQC	DeLuca et al.^87^	https://github.com/getzlab/rnaseqc
DESeq2	Love et al.^88^	https://bioconductor.org/packages/release/bioc/html/DESeq2.html
Apeglm	Zhu et al.^89^	https://bioconductor.org/packages/release/bioc/html/apeglm.html
clusterProfiler	Yu et al.^90^	https://bioconductor.org/packages/release/bioc/html/clusterProfiler.html
RAVEN2.0 toolbox	Wang et al.^91^	https://github.com/SysBioChalmers/RAVEN
Task-driven Integrative Network Inference for Tissues	Agren et al.^45^	https://github.com/SysBioChalmers/RAVEN
COnstraint-Based Reconstruction and Analysis (COBRA) Toolbox (v2.0)	Heirendt et al.^92^	https://opencobra.github.io/cobratoolbox/stable/index.html
MEMOTE	Lieven et al.^47^	https://memote.io/
Prism 9.0	GraphPad Software	https://www.graphpad.com

### Resource availability

#### Lead contact

Further information and requests for resources and reagents should be directed to and will be fulfilled by the lead contact, Dimitrios Anastasiou (dimitrios.anastasiou@crick.ac.uk).

#### Materials availability

This study did not generate new unique reagents.

### Experimental model and subject details

#### Mouse experiments

All the experimental procedures were conducted in conformity with public health service policy on humane care and use of laboratory animals, approved by The Francis Crick Institute’s Animal Welfare and Ethical Review Body (AWERB) and comply with a license to DA’s lab ratified by the UK Home Office. C57BL/6J mice were housed under a light-dark cycle of 12:12h with controlled temperature (22-24°C). Two-week old male mice were injected intraperitoneally with 25mg/kg of the carcinogen diethylnitrosamine (DEN). From the time of weaning, mice were fed a western-like diet (WD, TestDiet, AIN-76A, Table S1). Separate cohorts that were not injected with DEN (nonDEN) and cohorts provided a control diet (TestDiet, AIN-93G, Table S1) served as controls. Hence, the following experimental groups were generated: DEN^WD^, DEN^CD^, nonDEN^WD^, nonDEN^CD^. Caloric content of lipids and carbohydrates in WD is similar to average European diet.^31^

### Method details

#### Monitoring of tumour development with magnetic resonance imaging (MRI)

All *in vivo* MRI studies were performed on a 9.4 Tesla horizontal magnet (Biospec, Bruker, Germany). Mice were anaesthetized with 1-2% isoflurane in O_2_ and prone-positioned inside a^1^H quadrature volume coil (Bruker, Germany). The body temperature was maintained at 37 °C using a heating pad and breathing was monitored using a pressure sensor recording thorax movement (SA Instruments Inc, New York, USA). Tumor development was monitored regularly from 20 to 36 weeks in DEN-WD and DEN-CD mice. Axial and coronal MR images were acquired using breath-gated FLASH sequence with repetition time (TR) of 321ms, echo-time (TE) of 4.39ms, matrix of 256x256 and 4 averages.^93^

#### mRNA extraction, library preparation and sequencing

At the indicated experimental time points, mice were culled, livers were rapidly excised, tumors (where existing) were separated from adjacent tissue, and tissues were snap-frozen in liquid nitrogen. Samples were stored at -80°C. Total tissue was pulverised with mortar and pestle under a liquid nitrogen atmosphere and RNA was extracted from the equivalent of 20-30mg frozen tissue in TRIzol Reagent (Thermo Fisher Scientific, UK) followed by phenol removal with chloroform. RNA was further purified with the RNeasy Mini Kit (Qiagen, UK). DNase treatment was performed to remove any genomic DNA contamination. After RNA quantification and quality controls for integrity and purity (Nanodrop, Qubit and Agilent 2100 Bioanalyzer), libraries were prepared using KAPA mRNA HyperPrep Kit (Kapa Biosystems). mRNA sequencing (single-end, 40 million reads total) was performed on an Illumina HiSeq 2500 instrument.

#### Respiratory exchange ratio (RER) measurements of mice in metabolic cages

Eight DEN^WD^ and eight nonDEN^WD^ mice were individually housed in a laboratory animal monitoring system (TSE Phenomaster, TSE Systems GmBH). After an acclimatization period, O_2_ consumption and CO_2_ production, food, water intake and activity were continuously monitored for each mouse for a period of ≥48h. The mean O_2_ consumption and CO_2_ production rates measured over 48h were used to constrain context-specific GSMMs – see generation of context-specific GSMMs (csGSMMs) from [MMRNHep] for further details.

#### Processing of RNA-sequencing data

Raw RNA-sequencing data were processed using an in-house analysis pipeline. Quality of raw sequencing data was checked with FastQC v0.11.7 (http://www.bioinformatics.babraham.ac.uk/projects/fastqc). Reference genome alignment was performed against Genome Reference Consortium Mouse Build 38 (GRCm38) with RSEM^84^ and STAR.^85^ RSEM was used to generate raw counts, fragments per kilobase million (FPKM) and transcripts per million (TPM) which were used for all downstream analyses. Quality control metrics were reported with picard, RSeQC^86^ and RNA-SeQC.^87^ A final quality control report was generated with MultiQC.^94^ RNA-Seq data are available through the National Center for Biotechnology Information Gene Expression Omnibus (NCBI GEO, https://www.ncbi.nlm.nih.gov/geo/) accession number GSE199899.

#### Differential gene expression and enrichment analysis

The RSEM gene count matrix was used for differential gene expression (DGE) analysis with DESeq2^88^ with the apeglm algorithm for log-fold change shrinkage.^89^ Unless otherwise specified genes with an absolute minimum log-fold change of 1 and FDR-adjusted p-value ≤0.05 were considered statistically significant. The *clusterProfiler* package was used for Gene Ontology (GO) over-representation tests using the *enrichGO* function and Benjamini-Hochberg method was used to correct for multiple tests.^90^^,^^95^

#### Reconstruction of Mouse Metabolic Reaction Network (MMRN)

To generate a new mouse GSMM with increased metabolic network coverage, we used two human GSMMs, the Human Metabolic Reactions (HMR) database version 2 (HMR2)^36^ and Recon3D^35^ as templates. The protocol described here used known mouse gene orthologs of human genes as well as new gene orthologs identified by the homology of the protein sequences they encode to sequences of human proteins.

Human-to-mouse orthologs were downloaded using the online BioMart tool (www.biomart.org/, date accessed – January 2019). HMR2 uses Ensembl gene identifiers as gene annotations and human genes in HMR2 could therefore be directly replaced with their corresponding mouse ortholog.

To augment this network, two networks based on sequence homology between mouse and human protein sequences in HMR2 and Recon3D were reconstructed in a two-step process using the RAVEN2.0 toolbox^91^ implemented in MATLAB R2019b. In the first step, the *getBlast* function was used to perform a bi-directional BLASTP between the amino acid sequences of proteins in GRCm38 and the amino acid sequences of proteins encoded by genes in the HMR2 and Recon3D databases, respectively. The two resulting BLAST structures were then used as input for the *getModelFromHomology* function in the second step that aims to replace human genes within the reference GSMM with corresponding mouse orthologues provided that there is sufficient sequence alignment between genes (e-value cut-off 10^-30^).

A metabolite identifier map between HMR2 and Recon3D was generated (Table S2) and used to rename all metabolite identifiers in IM1-IM3 and the Mouse Metabolic Reaction (MMR)^26^ database to their corresponding KEGG identifiers. Universal metabolite nomenclature allowed step-wise integration of orthology reconstructions and MMR by merging the stoichiometric (S) matrices with the *mergeModels* function in RAVEN2.0.

The resulting reconstruction, Mouse Metabolic Reaction Network (MMRN), was computationally evaluated. Firstly, duplicate reactions, genes and metabolites were removed and the biomass reaction from HMR2 was added to the reconstruction. The *checkTasks* function was then used to assess whether MMRN can perform 56 common metabolic growth tasks.^45^ Finally, elemental balance of all reactions was assessed for carbon, nitrogen, oxygen, sulfur or phosphorous, and all imbalanced reactions were removed in a stepwise manner while ensuring consistent biomass flux and fulfilment of metabolic growth tasks. Reactions that resulted in decreased biomass flux or failure of a task were manually curated for mass-balance.

#### Benchmarking of MMRN

Three mGSMMs, Mouse1, MMR and iMM1865, and three hGSMMs, Human1, HMR2 and Recon3D, were used as references to compare to and benchmark MMRN. The *getElementalBalance* function in RAVEN2.0 was used to calculate the mass balance of all metabolic reactions for carbon, nitrogen, oxygen, sulfur or phosphorous. Connectivity was assessed by converting each GSMM to a bipartite graph using the S-matrix and counting the frequency of components. MEMOTE (version 0.13.0) was then used to evaluate MMRN using the same parameter settings as.^34^ In short, the report snapshot function in MEMOTE was used to generate HTML files for each model containing the evaluation results (found in https://github.com/sysbiomelab/MMRNHep/tree/main/data/memote). As in,^34^ the following four tests were left out of the analysis because they do not contribute to the overall MEMOTE score and require excessively long computation time due to the use of flux variability analysis: *test_blocked_reactions*, *test_find_stoichiometrically_balanced_cycles*, *test_find_metabolites_not_produced_with_open_bounds,* and *test_find_metabolites_not_consumed_with_open_bounds*.

MMRN scored well for most MEMOTE consistency attributes (mass balance, charge balance and metabolite connectivity), but, similar to HMR2, Recon3D and MMR, MMRN failed ‘stoichiometric consistency’. We used the SInConsistentMetBool variable of the findStoichConsistentSubset function in COBRA^92^ and found that the following metabolites contributed to the stoichiometric inconsistency: HDL, LDL, TAG-extraction, VLDL, chylomicron, chylomicron remnant, cofactors and vitamins, fatty acid-uptake pool, lipid droplet, phospholipids extracellular pool, vitamin A derivatives, vitamin D derivatives, vitamin E derivatives.

#### Reconstruction of [MMRN^Hep^]

The maximum FPKM expression values across all mouse experimental conditions were calculated for genes in MMRN. This was used with 56 common metabolic growth tasks as input for the task-driven Integrative Network Inference for Tissues, tINIT, (Agren et al. 2014), to generate a generic hepatic GSMM,[MMRN^Hep^]. The expression of genes was classified as high (FPKM ≥50), medium (10 ≤ FPKM >50), low (1 ≤ FPKM <10) or no expression (FPKM <1).

#### Generation of context-specific GSMMs (csGSMMs) from [MMRN^Hep^]

##### Gene expression-based constraining

An adapted version of the E-flux^46^ method was used to construct constraint vectors for the lower- and upper reaction bounds of [MMRN^Hep^]. For this purpose, a vector *b* was constructed for each experimental condition using expression data and gene-reaction rules (GR-rules) of [MMRN^Hep^] as follows. For a reaction catalyzed by a single gene the mean expression value across biological replicates was considered for *b*_*j*_. For reactions with multiple enzymes associated with ‘or’ relationships the sum of the mean expression values for individual genes was considered for *b*_*j*_. For reversible reactions the negative value of *b*_*j*_ was also imposed as a lower bound allowing these reactions to be bi-directional. Orphan reactions, reactions without gene association, were kept unconstrained.

##### O_2_ and CO_2_ constraining

We used the RER measured for DEN and nonDEN mice to constrain oxygen uptake and carbon dioxide production for DEN and nonDEN GSMMs, respectively (Table S5). For this purpose, the mean volumes of oxygen consumption and CO_2_ production for 5 mice were converted to flux values (mmol/mouse/day) using Equation 1

(Equation 1) $$b=\frac{pV}{M}$$

where *b* is the constraint value, *p* the density, *V* the average volume and *M* the molecular weight of either oxygen or carbon dioxide. The calculated flux values were used to set the lower bound of corresponding reactions.

##### Diet-based constraining

The content of the WD and the CD was used to constrain uptake rates (or exchange reactions) of the GSMM using Equation 2

(Equation 2) $$b=\frac{PW}{M}$$

where *b* is the constraint value, *P* is the % w/w of a metabolite in the diet, *M* the molecular weight of that metabolite and *W* the average grams of food consumed per mouse per day (3g). The % w/w content of nutrients in CD and WD was obtained from the respective diet information sheets, AIN-93G and AIN-76A (see *Mouse experimental design*) and also listed in Table S6. We divided the stated diet content for saturated FAs equally between palmitate, stearate, myristic acid and lauric acid. Similarly, we divided the stated content for monounsaturated FAs equally among eicosenoic acid, octadecenoic acid and palmitoleic acid.

The calculated values (mmol/mouse/day) were used to constrain the upper bounds exchange reactions and thus reflect the maximum uptake rate of a particular metabolite available to the GSMM to perform flux balance analysis. The carbon flux (or *C*_*moles*_) for each diet was calculated using Equation 3

(Equation 3) $$C_{moles}=\sum_{j=1}^{n}C\;b_{j}$$

where *C*_*moles*_ is the moles of carbons, *C* the number of carbons in each metabolite and *b* the upper bound flux for *j* = 1 to *n* metabolites within each diet. The same equation was used to calculate the *C*_*moles*_ for uptake by using the calculated uptake flux value, *v*, instead of *b* for each metabolite. Defining the physiologically correct metabolic input for flux simulations in mammalian tissue GSMMs is challenging^56^ and directly translating the diet to input flux remains the state of the art.^26^^,^^96^ Therefore, for the purpose of this analysis, it is assumed that components in the mouse diet are directly available to tissues.

#### Flux balance analysis (FBA) and differential flux analysis

The *solveLP* function in the RAVEN2.0 toolbox was used with the parameter to minimize the sum of all absolute fluxes using the MOSEK version 7 solver (www.mosek.com) in MATLAB R2019a. The HMR2 biomass equation was used as an objective function for all GSMMs. Since the chosen objective has a remarkable influence on the resulting flux distribution when solving the linear problem, we chose to use the same objective for all GSMMs to be able to do pairwise comparisons of all of flux vectors. To compare two flux distributions, we calculated the Euclidean distance between two flux vectors using the SciPy package implemented in python3.7. To identify differential flux reactions between two FBA experiments we directly compared two flux vectors and calculated a flux ratio between two experiments.

#### Isolation of mitochondria from liver and oxygen flux measurements

Mice were culled and the tissues of interest (liver, peritumoral liver and tumor) were quicky excised and rinsed in cold phosphate buffer before being transferred to mitochondrial isolation buffer (250mM sucrose, 10mM Hepes, 0.1% BSA fatty-acid free, pH 7.2). Tissue was homogenized on ice with a Potter-Elvehjem homogeniser before centrifugation at 800x*g* for 10 minat 4°C. After discarding the top lipid layer, the supernatant was centrifuged at 10000x*g* for 10 minat 4°C. The pellet was resuspended in washing buffer (250mM sucrose, 10mM Hepes pH7.2) and centrifuged again at 10000x*g* for 10 minat 4°C, a process that was repeated twice. The resulting mitochondrial pellet was resuspended in 0.5mL of washing buffer. Mitochondrial protein quantification was performed with Pierce BCA Protein assay.

Mitochondrial respiration driven by fatty acid oxidation was assessed with an oxygen electrode system (Oroboros Oxygraph-2K, Oroboros Instruments, Austria) using 1mg of mitochondrial protein in 2mL of working buffer (130mM sucrose, 50mM KCl, 5mM KH_2_PO_4_, 5mM MgCl_2_, 5mM Hepes, 50μM EDTA, pH7.2 at 37°C) supplemented with 4mM ADP and 0.5mM octanoyl-carnitine.

#### *De novo* lipogenesis (DNL) assessed with ^2^H_2_O

The contribution of *de novo* lipogenesis (DNL) to hepatic or tumoral triglyceride (TAG) pool was determined *in vivo* using ^2^H_2_O.^93^^,^^97^ In brief, mice were intraperitoneally injected with ^2^H_2_O and supplied with 5% ^2^H_2_O drinking water while feeding *ad libitum*. 16 h later, animals were culled, tissues were collected and frozen in liquid N_2_. Tissues were powdered under liquid N_2_ atmosphere and lipids were extracted with the Folch method.^98^ Triglycerides were isolated using ISOLUTE® NH2 SPE (Biotage, UK). *De novo* synthesised FAs and glycerol incorporated in TAGs were determined as the fraction of FA methyl or glyceryl moiety, respectively, in TAG that were labeled with ^2^H, normalized for the enrichment of ^2^H in H_2_O in the plasma of each mouse measured using ^2^H NMR.^99^

### Quantification and statistical analysis

Statistical analysis for RNA-sequencing data was performed in R software v 4.1.2 using the DESeq2 package; genes with an FDR-adjusted p-value ≤0.05 and absolute log_2_-fold change ≥1 were considered statistically significant. Statistical analysis of all experimental procedures was performed in GraphPad Prism software v9.0. The specific statistical tests used are described in the corresponding figure legends.

## Data Availability

• RNA-sequencing data have been deposited at GEO and are publicly available as of the date of publication. Accession numbers are listed in the key resources table. • All original code has been deposited at Zenodo and is publicly available as of the date of publication. The DOI is listed in the key resources table. • Any additional information required to reanalyse the data reported in this paper is available from the lead contact upon request.

## Supplemental information

Document S1. Figures S1–S4

Table S1. Contributions of major dietary components (amino acids, carbohydrates and lipids) to western (WD) and control (CD) diet composition, related to Figure 1 and STAR Methods

Table S2. Metabolite identifier map used to rename metabolites in IM1-4, related to Figure 2A and STAR Methods

Table S3. Summary of MEMOTE scores for MMRN and other published mouse and human GSMMs shown in Figure S2B, related to Figure 2 and STAR Methods

Table S4. Key metabolic network attributes for MMRN compared to other published mouse and human GSMMs shown in Figure S2B, related to Figure 2 and STAR Methods

Table S5. Oxygen uptake and carbon dioxide production for DEN and nonDEN animals, related to STAR Methods

Table S6. Conversion of amino acid, carbohydrate and lipid content in mouse diet to flux values, and calculated nutrient uptake fluxes for different csGSMMs, related to Figures 3 and S3, and STAR Methods

Table S7. Flux distributions for 5 csGSMMs on two diets as well as diet component swaps performed using SyDiCoS, related to Figure 3

Table S8. Euclidean distances calculated between flux distributions for different dietary inputs, related to Figures 4A–4C

Table S9. Fluxes in tumor and peritumoral tissue models for subsystems identified in Figure 4D and used for Figures 4E, 4F, S4B, and S4C

Table S10. Differential gene expression for genes associated with reactions in Figures 4E, 4F, and S4

Data S1. Cytoscape file showing of all reactions in subsystems that have a reaction ratio = 1 (either in [MMRN^Hep^]^DEN−T-WD^ or in [MMRN^Hep^]^DEN−PT-WD^) with WD (Figure 4D, left) and partake in lipid and carbohydrate metabolism, related to Figure 4D Reaction identifiers are indicated by diamond-shaped nodes and can be colored with the table loaded in the file for different FBA experiments described in the text [WD: full WD, CD: full CD, CARB: WDcarbs(CD), LIPID: WDlipids(CD)]. The labels (“higher”, “lower”, “equal”, “noFlux”) for these nodes refer to flux differences in T relative to PT.
