# Supplementary material for: Systematic diet composition swap in a mouse genome-scale metabolic model reveals determinants of obesogenic diet metabolism in liver cancer
Source: iScience. 2023 Jan 24;26(2):106040. doi: 10.1016/j.isci.2023.106040 (PMC9947310; doi:10.1016/j.isci.2023.106040)
Supplement: Document S1. Figures S1–S4 [file mmc1.pdf]

iScience, Volume *26*

Supplemental information

Systematic diet composition swap in a mouse genome-scale metabolic model reveals determinants of obesogenic diet metabolism in liver cancer

Frederick Clasen, Patrícia M. Nunes, Gholamreza Bidkhori, Nourdine Bah, Stefan Boeing, Saeed Shoaie, and Dimitrios Anastasiou

SUPPLEMENTAL FIGURES

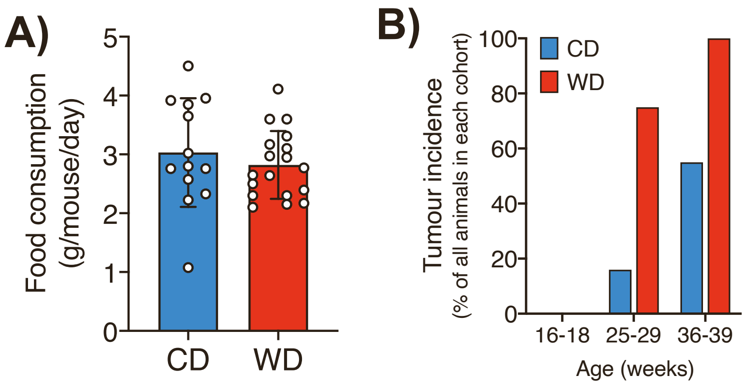

Figure S1. Food consumption and tumour incidence measurements for mice used in experiments related to Figure 1.
A) Food consumption measurements for mice fed a CD or WD used to calculate C_{moles}^{DIET} in this study. Data are represented as mean ± SD.
B) The tumour incidence for mice fed either a CD or WD at different age intervals. At time of tissue harvesting (36-39 weeks of age) all WD mice had tumours compared to 55% of mice with tumours on a CD.

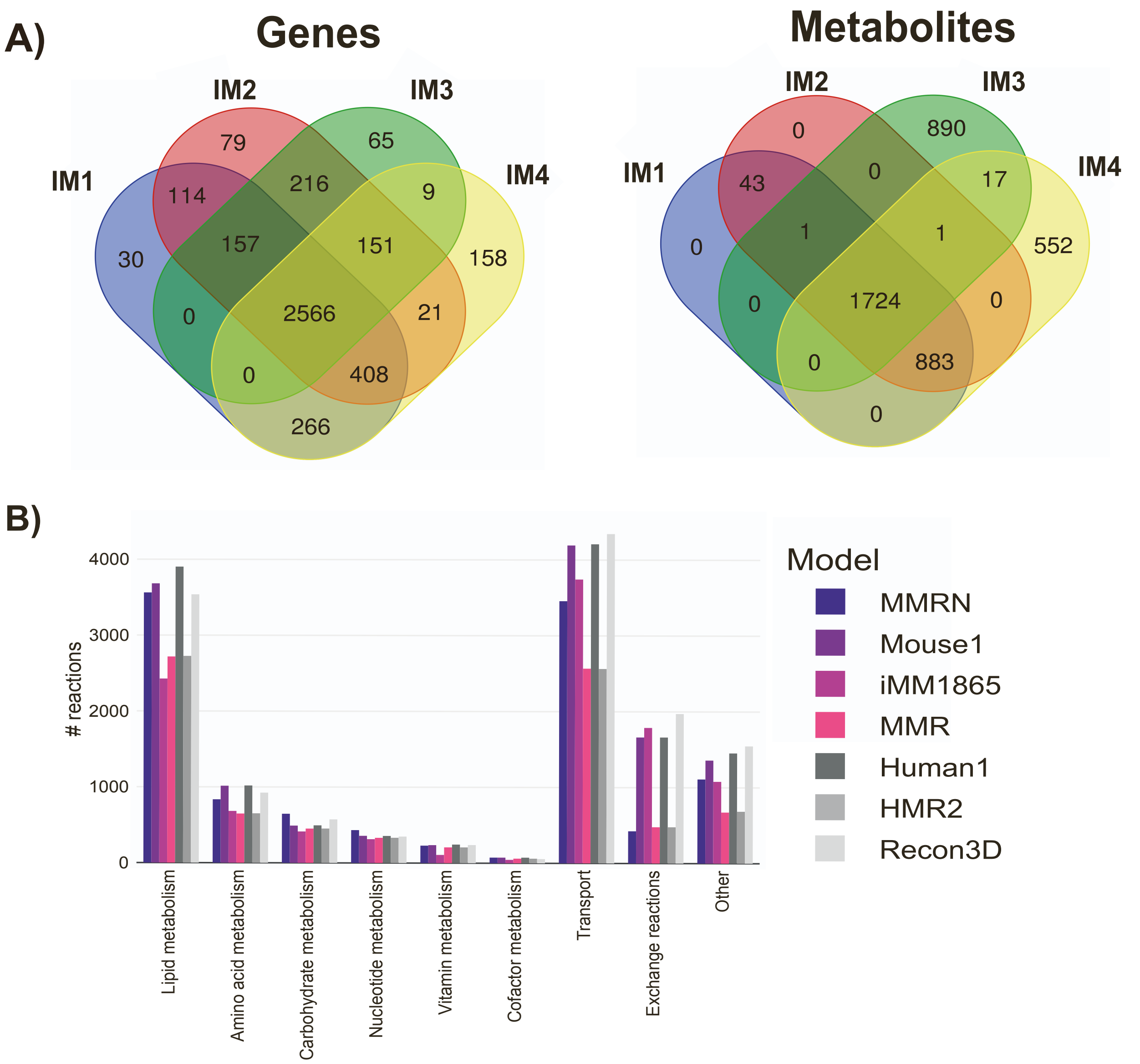

Figure S2. Model attributes linked to MMRN construction and benchmarking (related to Figure 2).
A) IM1-4 used to reconstruct MMRN are not redundant. Overlap of genes and metabolites for IM1-4 shown as Venn diagrams.
B) Number of reactions in MMRN compared to previously published mouse GSMMs Mouse1 [1], MMR [2], iMM1865 [3] and human GSMMs Human1 [4], HMR2 [5], Recon3D [6].

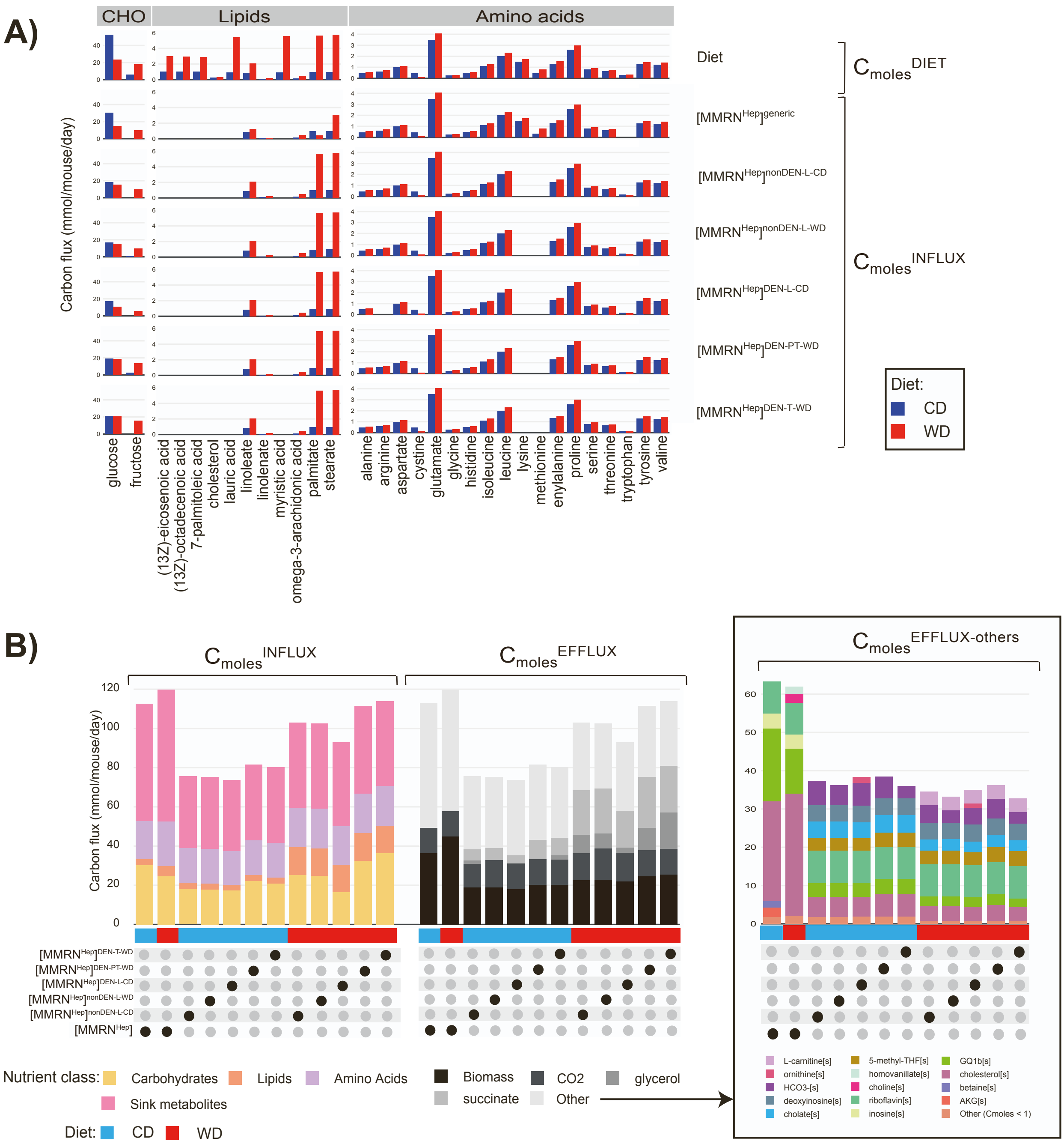

Figure S3. Breakdown of C_{moles} values related to data presented in Figure 3.
A) C_{moles}^{DIET} (top) and C_{moles}^{INFLUX} for individual dietary nutrients that belong to the three major dietary component classes (carbohydrates, lipids and amino acids) for CD and WD.
B) C_{moles}^{INFLUX} (left panel) for different csGSMMs as in **Figure 3B**, but including metabolites such as co-factors, vitamins and oxygen that partake in sink reactions and are added to ensure functionality of the model. The C_{moles}^{EFFLUX} equals the C_{moles}^{INFLUX} values for each model when the sink reactions are included, attesting to the carbon balance of [MMRN^{Hep}]. The right panel depicts the C_{moles}^{EFFLUX} of metabolites that do not fall under the three major nutrient class categories (carbohydrates, lipids, amino acids). This graph illustrates that increases in the C_{moles}^{EFFLUX} values of glycerol and succinate largely account for the increase in total C_{moles}^{EFFLUX} in WD compared to CD.

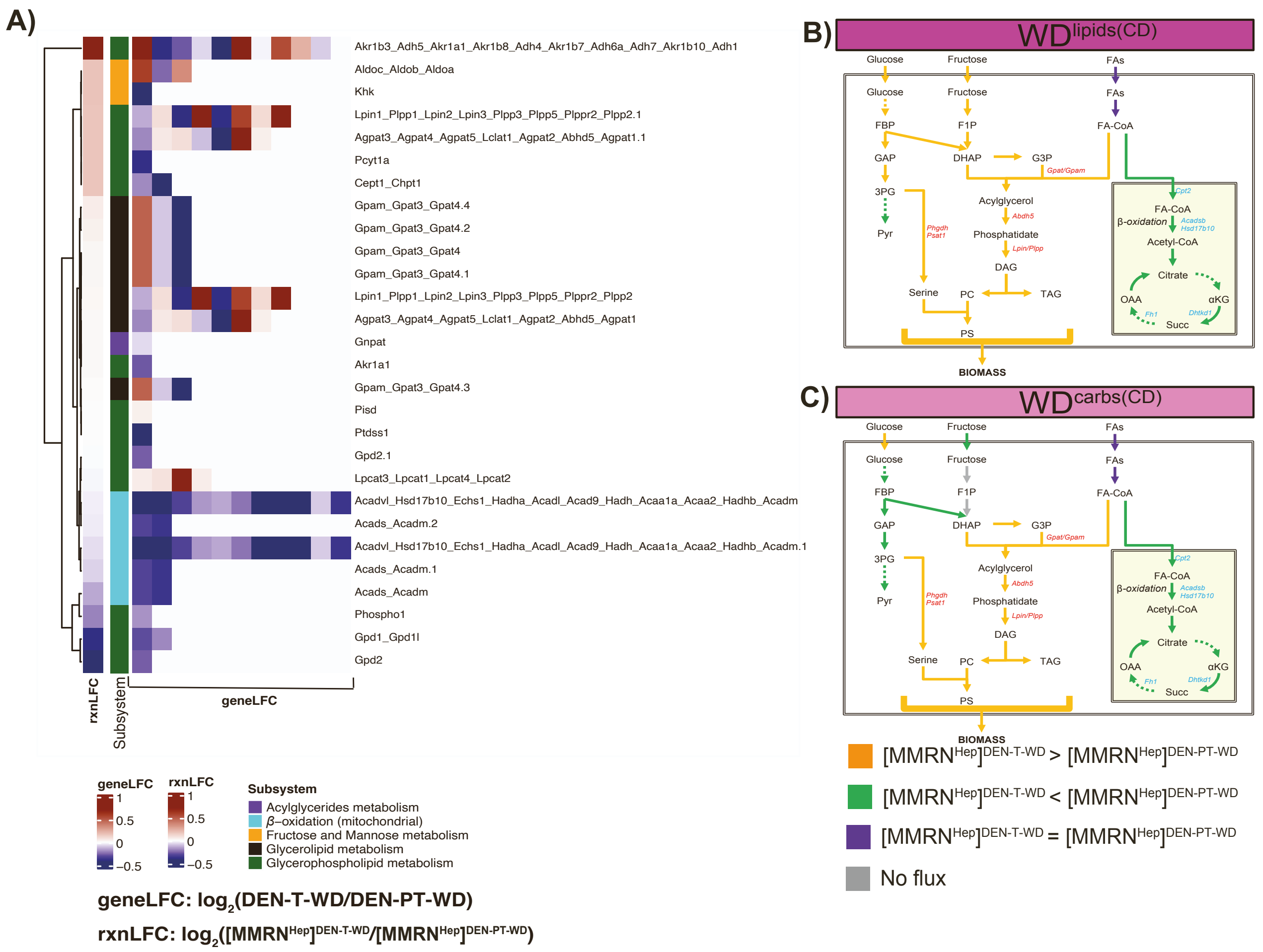

Figure S4. Differences in measured gene expression and in estimated fluxes between T and PT tissues related to Figure 4.

A) Log2-fold changes of gene expression and reaction fluxes shown in **Figure 4E**. The log2-fold change in the expression of each gene associated with the corresponding reaction is plotted for DEN^{T} compared to DEN^{PT}.

B) Metabolic network as in **Figure 4E** showing lux differences in tumoral and peritumoral models in $WD^{lipids(CD)}$.

C) Metabolic network as in **Figure 4E** showing lux differences in tumoral and peritumoral models in $WD^{carbs(CD)}$.

SUPPLEMENTAL REFERENCES

S1. Wang, H., Robinson, J.L., Kocabas, P., Gustafsson, J., Anton, M., Cholley, P.E., Huang, S., Gobom, J., Svensson, T., Uhlen, M., et al. (2021). Genome-scale metabolic network reconstruction of model animals as a platform for translational research. Proc Natl Acad Sci U S A *118*. 10.1073/pnas.2102344118.

S2. Mardinoglu, A., Shoaie, S., Bergentall, M., Ghaffari, P., Zhang, C., Larsson, E., Backhed, F., and Nielsen, J. (2015). The gut microbiota modulates host amino acid and glutathione metabolism in mice. Mol Syst Biol *11*, 834. 10.15252/msb.20156487.

S3. Khodaee, S., Asgari, Y., Totonchi, M., and Karimi-Jafari, M.H. (2020). iMM1865: A New Reconstruction of Mouse Genome-Scale Metabolic Model. Sci Rep *10*, 6177. 10.1038/s41598-020-63235-w.

S4. Robinson, J.L., Kocabas, P., Wang, H., Cholley, P.E., Cook, D., Nilsson, A., Anton, M., Ferreira, R., Domenzain, I., Billa, V., et al. (2020). An atlas of human metabolism. Sci Signal *13*. 10.1126/scisignal.aaz1482.

S5. Mardinoglu, A., Agren, R., Kampf, C., Asplund, A., Uhlen, M., and Nielsen, J. (2014). Genome-scale metabolic modelling of hepatocytes reveals serine deficiency in patients with non-alcoholic fatty liver disease. Nat Commun *5*, 3083. 10.1038/ncomms4083.

S6. Brunk, E., Sahoo, S., Zielinski, D.C., Altunkaya, A., Drager, A., Mih, N., Gatto, F., Nilsson, A., Preciat Gonzalez, G.A., Aurich, M.K., et al. (2018). Recon3D enables a three-dimensional view of gene variation in human metabolism. Nat Biotechnol *36*, 272-281. 10.1038/nbt.4072.
